# Anomalous right coronary artery arising from the pulmonary artery and constrictive pericarditis: an unusual association

**DOI:** 10.1590/S1679-45082013000300018

**Published:** 2013

**Authors:** Odilson Marcos Silvestre, Eduardo Leal Adam, Dirceu Thiago Pessoa de Melo, Ricardo Ribeiro Dias, Felix J. A. Ramires, Charles Mady

**Affiliations:** 1 Instituto do Coração, Hospital das Clínicas, Faculdade de Medicina, Universidade de São Paulo, São Paulo, SP, Brazil.

**Keywords:** Pericarditis, Coronary disease, Heart failure, Case reports

## Abstract

The association of anomalous right coronary artery originating from the pulmonary artery and constrictive pericarditis has never been showed in the literature. We present the first case of this unusual association in a patient with right heart failure. After diagnosis, the patient was referred to surgery and underwent phrenic-to-phrenic pericardiectomy; graft implant of right internal thoracic artery to right coronary artery; and ligation of the anomalous origin of the right coronary artery from the pulmonary artery. Such procedures solved the potential risk of sudden death related to anomalous right coronary artery originating from the pulmonary artery and alleviated the symptoms of heart failure caused by constrictive pericarditis.

## RESUMO

A associação da artéria coronária direita anômala com origem na artéria pulmonar e pericardite constritiva ainda não foi descrita na literatura. Apresentamos aqui o primeiro caso dessa associação inusitada em um paciente com quadro de insuficiência cardíaca direita. Após o diagnóstico, o paciente foi encaminhado para tratamento cirúrgico, sendo submetido a pericardiectomia frênico a frênico, implante de enxerto da artéria mamária interna direita para a coronária direita e ligadura da origem anômala da coronária direita da artéria pulmonar. Tais procedimentos resolveram o potencial risco de morte súbita pela anomalia coronária e aliviaram os sintomas de insuficiência cardíaca causados pela pericardite constritiva.

## INTRODUCTION

Several heart diseases manifest as right-sided heart failure (HF), with lower limb edema, ascites, hepatomegaly, and jugular venous distention. Constrictive pericarditis is an uncommon etiology, often underdiagnosed, that holds importance for being a reversible cause of HF after surgical treatment^(1)^.

An anomalous right coronary artery originating from the pulmonary artery (ARCAPA) is a rare coronary anomaly, usually incidentally diagnosed, that has been associated to sudden cardiac death^(2,3)^. There are no reports to date of these two unrelated conditions in the same patient. The aim of this report was to describe the first case of this unusual association.

## CASE REPORT

A 24-year-old man developed lower limbs edema and exertion dyspnea in the past 3 years. When he first came to our institution, he was already receiving high doses of furosemide, hydrochlorothiazide and spironolactone, without an established diagnosis.

On physical examination, lower limb edema and stasis dermatitis were observed. The apical impulse was normally placed, and jugular venous distention was not present. Upon cardiac auscultation, wide inspiratory splitting of the second heart sound, and no other sounds or murmurs.

The electrocardiogram was normal. Chest radiography showed a normal cardiac silhouette, without calcifications or signs of pulmonary congestion. The brain natriuretic peptide (BNP) level was 69pg/mL (reference range <100pg/mL). Echocardiogram revealed normal function of right and left ventricles, biatrial enlargement, and an estimated pulmonary artery systolic pressure of 25mmHg. The pericardium was described as normal. Coronary arteries were dilated, and the left main coronary artery measured 7.6mm and the right coronary artery (RCA), 10mm.

**Figure 1:**
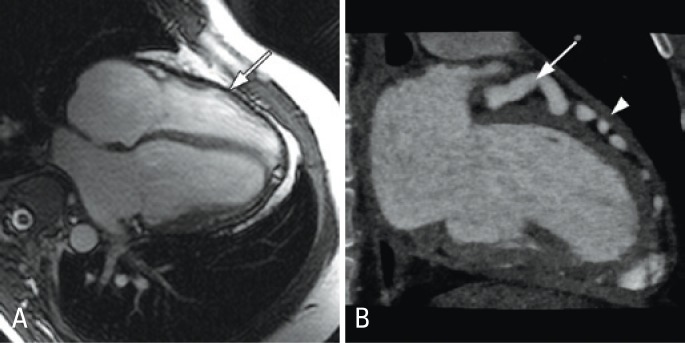
(A) Cardiac magnetic resonance imaging showing pericardial thickening (arrow). (B) Dilated left anterior descending artery (arrow) and thickened pericardium (arrowhead) as demonstrated by cardiac computed tomography

**Figure 2:**
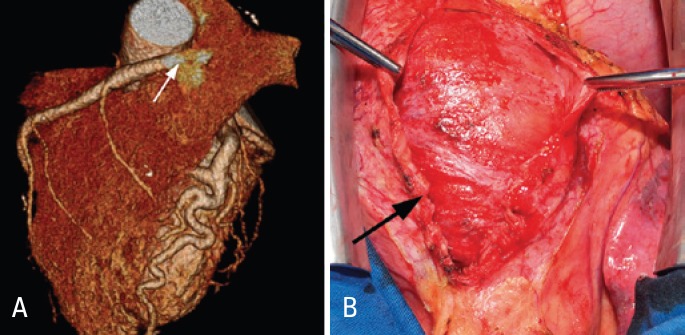
(A) Coronary computed tomography angiogram showing the anomalous right coronary artery originating from the pulmonary artery (white arrow). (B) Intraoperative view of the thickened pericardium (black arrow)

The diagnosis of constrictive pericarditis was considered due to the presence of symptoms and signs of HF with normal morphology and function of both ventricles. Magnetic resonance imaging of the heart showed a diffusely thickened pericardium and interventricular septal bounce during systole (Figure 1). Coronary computed tomography angiogram was performed to evaluate the unexpected coronary dilation observed on echocardiogram. An anomalous RCA arising from the pulmonary artery was diagnosed (Figure 2).

Surgical resection of the diseased pericardium was performed along with coronary revascularization, by performing a right internal thoracic artery graft to the RCA and occlusion of the anomalous origin of this vessel at the pulmonary artery. Pathological assessment of the pericardium demonstrated a nonspecific chronic pericarditis of low intensity. Polymerase chain reaction used to detect *Mycobacterium tuberculosis* was negative. The patient was discharged home with marked improvement of symptoms.

## DISCUSSION

The case presented describes a patient with symptoms of right HF secondary to constrictive pericarditis, with simultaneous diagnosis of an ARCAPA. It also highlights the importance of considering the diagnosis of constrictive pericarditis in the evaluation of right HF.

Constrictive pericarditis results from chronic inflammation of the pericardium, which becomes thickened and calcified, leading to diastolic dysfunction. Although most cases are idiopathic, it can be caused by previous cardiac surgery, following mediastinal radiation, due to connective tissue diseases and tuberculosis.

In patients with a constrictive picture, the thickened pericardial sac impairs ventricular dilation and filling during diastole, leading to an abrupt elevation of pressure in cardiac chambers. On inspiration, the rigid pericardium does not allow the right ventricle to accommodate the increased venous return, which leads to a bounce of the interventricular septum, a reduction in left ventricular cavity and a low cardiac output.

These physiologic derangements may manifest as jugular venous distention, prominent venous Y waves, ascites, lower limb edema and Kussmaul's sign. The pericardial knock, an early diastolic sound of high frequency, is diagnostic of constrictive pericarditis but it is present in only one third of cases. Previous studies showed low accuracy of physical examination for diagnosis of constrictive pericarditis. In the case reported, many physical findings suggestive of the condition were not seen, in part due to the high doses of diuretics the patient was receiving.

The chest radiograph may reveal a pericardial calcification in up to 30% of patients. Echocardiogram is useful to rule out other causes of right HF, but has limited capacity to evaluate the pericardium and is often inconclusive^(1)^. Laboratory assessment of BNP levels may help in the differential diagnosis of constrictive pericarditis with restrictive cardiomyopathies^(4)^. In the former, BNP levels are usually not elevated, as observed in this case, because myocardial stretch is limited by the pericardium, while in the latter there is significant myocardial wall stretch, and BNP levels can be strikingly increased.

Cardiac magnetic resonance imaging is the gold standard for the noninvasive diagnosis of constrictive pericarditis. It clearly demonstrates the degree of pericardial thickening and the ventricular interdependence with septal bouncing. It is also of extreme value for excluding other conditions affecting the myocardium.

Many reports demonstrated good outcomes after surgical treatment of constrictive pericarditis. While surgical mortality remains around 6 to 12%, the 5-year survival rate without symptoms of severe HF is 80%^(5)^. Therefore, making the diagnosis of this condition is extremely important for providing appropriate surgical care and changing the natural history of this progressively debilitating disease.

The ARCAPA is a congenital defect found in 0.002% of the population. It represents 0.12% of coronary anomalies. Its natural history is not welldefined, but in most cases it was incidentally diagnosed in asymptomatic patients^(2)^. In symptomatic patients, clinical features may include exertional chest pain, syncope, sudden cardiac death, or findings of ischemic cardiomyopathy. The mechanism of sudden cardiac death is not completely understood, but may be related to the presence of ischemia in the myocardial territory supplied by the RCA, due to insufficient collaterals from the left anterior descending and left circumflex arteries. Coronary ectasia, a frequent finding in patients with ARCAPA, is secondary to increased blood flow through collaterals from the left coronary artery into the RCA, followed by reverse flow in the RCA into the pulmonary trunk. In the present report, the symptoms of right HF were caused by the pericardial pathology and were probably unrelated to the coronary malformation. Sudden cardiac death has been linked to ARCAPA^(3)^, and therefore surgical correction is the preferred treatment even in asymptomatic patients. Since the patient was undergoing pericardiectomy, correction of the coronary anomaly was performed in the same procedure.

In conclusion, the case described herein characterizes constrictive pericarditis as a cause of HF of difficult diagnosis, and also describes the presence of a rare coronary anomaly in the same patient. Two uncommon and unrelated diseases were successfully treated in the same surgical procedure.
